# Dorsal switch protein 1 as a damage signal in insect gut immunity to activate dual oxidase *via* an eicosanoid, PGE_2_

**DOI:** 10.3389/fimmu.2022.994626

**Published:** 2022-11-10

**Authors:** Miltan Chandra Roy, Shabbir Ahmed, Yonggyun Kim

**Affiliations:** Department of Plant Medicals, Andong National University, Andong, South Korea

**Keywords:** insect, immunity, gut, DSP1, eicosanoid, PGE2

## Abstract

Various microbiota including beneficial symbionts reside in the insect gut. Infections of pathogens cause dysregulation of the microflora and threaten insect survival. Reactive oxygen species (ROS) have been used in the gut immune responses, in which its production is tightly regulated by controlling dual oxidase (Duox) activity *via* Ca^2+^ signal to protect beneficial microflora and gut epithelium due to its high cytotoxicity. However, it was not clear how the insects discriminate the pathogens from the various microbes in the gut lumen to trigger ROS production. An entomopathogenic nematode (*Steinernema feltiae)* infection elevated ROS level in the gut lumen of a lepidopteran insect, *Spodoptera exigua*. Dorsal switch protein 1 (DSP1) localized in the nucleus in the midgut epithelium was released into plasma upon the nematode infection and activated phospholipase A_2_ (PLA_2_). The activated PLA_2_ led to an increase of PGE_2_ level in the midgut epithelium, in which rising Ca^2+^ signal up-regulated ROS production. Inhibiting DSP1 release by its specific RNA interference (RNAi) or specific inhibitor, 3-ethoxy-4-methoxyphenol, treatment failed to increase the intracellular Ca^2+^ level and subsequently prevented ROS production upon the nematode infection. A specific PLA_2_ inhibitor treatment also prevented the up-regulation of Ca^2+^ and subsequent ROS production upon the nematode infection. However, the addition of PGE_2_ to the inhibitor treatment rescued the gut immunity. DSP1 release was not observed at infection with non-pathogenic pathogens but detected in plasma with pathogenic infections that would lead to damage to the gut epithelium. These results indicate that DSP1 acts as a damage-associated molecular pattern in gut immunity through DSP1/PLA_2_/Ca^2+^/Duox.

## Introduction

Wild animals including insects are heterotrophs and ingest nutrients for their survival. Diets containing nutrients cannot avoid contamination with various microbes. To protect against any pathogenic microbial infection during ingestion, insects have cuticle structures in the integument of the foregut and hindgut (1). In contrast, the insect midgut lacks the cuticle barrier, and thus it is relatively susceptible to microbial pathogens (2). To effectively defend the pathogen infection, the midgut possesses a peritrophic matrix as a physical barrier and also harbors commensal or mutualistic microbes for detoxifying xenochemicals or defending against other pathogens in addition to assisting digestion by supplementing essential nutrients (3, 4). In addition to this indirect defense machinery, insect midgut performs direct antimicrobial activities by producing reactive oxygen species (ROS) and antimicrobial peptide (AMP) (5, 6). ROS and AMP may cause self-damage to insects and should be tightly regulated in their production so as to selectively kill pathogenic microbes while keeping beneficial microbes (7, 8). For example, peptidoglycans derived from beneficial enterobacteria can trigger AMP expression, in which Caudal, a homeobox protein, can selectively repress the unnecessary expression of IMD/NF-kβ-dependent AMP genes in the midgut (9). However, upon pathogenic microbial infection, the midgut produces ROS to remove the pathogens (10).

Upon infection by pathogenic microbes, the midgut epithelium produces ROS by the catalytic activity of dual oxidase (Duox) (11). There are at least two ways to up-regulate ROS production by Duox. One is to elevate the Ca^2+^ level to activate Duox enzyme activity (12). The other is to induce *Duox* expression, which is regulated by uracil released from pathogens in *Drosophila* (7). The binding of uracil to an unidentified receptor on the midgut epithelial membrane triggers PLCβ/PKC/Ca^2+^ signal pathway to activate Duox (13). The hedgehog signaling pathway modulates uracil-induced Duox activation by forming Cadherin 99C-dependent endosomes as a platform for subsequent activation (13). In contrast, Duox of a lepidopteran *Spodoptera exigua* is activated by eicosanoids (14). Inhibition of eicosanoid biosynthesis prevents ROS production of the midgut upon pathogen infection (14). In the midgut of another lepidopteran *Plutella xylostella*, Duox gene expression and subsequent ROS production are essential for defending against bacterial pathogens (15). These results suggest that eicosanoids can mediate ROS production by activating Duox in the lepidopteran insects. Eicosanoids are a group of oxygenated C20 polyunsaturated fatty acids (PUFAs) that can mediate various physiological processes including immune responses in animals (16). Eicosanoid production is initiated by the release of free C20 PUFA from phospholipids by the catalytic activity of phospholipase A_2_ (PLA_2_) (17). Among eicosanoids, prostaglandins (PGs) play a crucial role in mediating various immune responses in the midguts of lepidopteran and dipteran insects (15, 18). In particular, several PGs along with the PGE_2_ receptor and its downstream signal components have been found in *S. exigua* and mediate the induction of intracellular Ca^2+^ levels (19). Thus, PGE_2_ is likely to mediate ROS production by inducing Duox activity in *S. exigua* by elevating the Ca^2+^ level through the PGE_2_ receptor.

Although uracil is specifically produced by pathogenic bacteria in the midgut of *Drosophila* (7), it is not clear that it also plays a crucial role in acting as a pathogen motif in various microbial pathogens including entomopathogenic viruses, nematodes, or fungi in the insect midgut. Here we propose a novel insect damage-associate molecular pattern (DAMP), which triggers gut immunity upon pathogen infection. Dorsal switch protein 1 (DSP1) is known as an insect DAMP molecule (20). DSP1 is an orthologue of vertebrate high mobility group B1 (HMGB1) (21, 22). HMGB1 is a ubiquitously expressed and highly conserved nuclear protein that plays important roles in chromatin organization and transcriptional regulation in mammals (23). HMGB1 consists of two HMG boxes (box A and box B) and a long acidic tail (24, 25). These two HMG boxes have different functions and properties: box A recognizes pre-bent and linear DNAs while box B binds to mini-circular DNA or bending linear DNA (26, 27). Upon stress conditions including pathogen infection, HMGB1 is released and acts as a DAMP to activate the innate immune system by interacting with pattern recognition receptors (28). Similarly, insect DSP1 acts as a transcription factor as well as a chromatin remodeling factor (22, 29). In *S. exigua* infected by entomopathogenic bacteria, DSP1 is released from fat body and activates PLA_2_ to elevate eicosanoids, which mediate cellular and humoral immune responses (20). DSP1 of a coleopteran insect, *Tenebrio molitor* also mediates the immune responses to bacterial infection (30).

This study investigated the DAMP role of DSP1 in gut immunity of *S. exigua* using an entomopathogenic nematode, *Steinernema feltiae*. To test this hypothesis, we monitored ROS production of the midgut after oral infection of the nematode. In the meantime, DSP1 was assessed in its release and mediation of Ca^2+^ signal to induce Duox activity to produce ROS.

## Materials and methods

### Insect rearing

All experiments in this study were conducted with *S. exigua* larvae originating from Welsh onion (*Allium fistulosum* L.) fields in Andong, Korea, and reared in a laboratory under 25°C with 16:8 h (L:D) and 60 ± 5% relative humidity. Five instar (‘L1-L5’) larvae were reared on an artificial diet and larval instar was determined by head capsule size (31). Adults were supplied with a 10% sucrose solution.

### Chemicals

Arachidonic acid (AA: 5,8,11,14-eicosatetraenoic acid), dexamethasone [DEX: (11β, 16α)-9-fluoro-11,17,21-trihydroxy-16-methylpregna-1,4-diene-3], naproxen [NAP: (S)-(+)-2-(6-methoxy-2-naphthyl)propionic acid], esculetin (ESC: 6-hydroxy-7-methoxycoumarin), 3-ethoxy-4-methoxyphenol (EMP) were purchased from Sigma-Aldrich Korea (Seoul, Korea) and dissolved in dimethyl sulfoxide (DMSO). Leukotriene B4 (LTB4: 5S,12R-dihydroxy-6Z,8E,10E,14Z-eicosatetraenoic acid) was purchased from Cayman Chemicals (Ann Arbor, MI, USA) and dissolved in DMSO. Prostaglandin E2 (PGE2: (5Z,11α,13E,15S)-11,15-dihydroxy-9-oxoprosta-5,13-dienoic acid), prostaglandin D2 (PGD2: 9α,15S-dihydroxy-11-oxoprosta-5Z,13E-dien-1-oic acid), 2-aminoethoxydiphenylborate (2-APB), dantrolene sodium (DAN: 1-[(5-(p-nitrophenyl) furfurylidene)amino] hydantoin sodium salt), and U-73122 (1-[6-[((17β)-3-methoxyestra-1,3,5[10]-trien-17-yl)amino]hexyl]-1H-pyrrole-2,5-dione) were purchased from Sigma-Aldrich Korea and dissolved in DMSO. Fura-8AM was purchased from AAT Bioquest (Sunnyvale, CA, USA) and dissolved in DMSO. Paraquat (1,1′-dimethyl-4,4′-bipyridinium dichloride hydrate) and vitamin C (L-ascorbic acid) were purchased from Sigma-Aldrich Korea and dissolved in distilled water. Phosphate-buffered saline (PBS) was prepared with 100 mM phosphoric acid and adjusted to pH 7.4.

### Nematode rearing

Infective juveniles (IJs) of *S. feltiae* were donated from a laboratory strain maintained in Nambo, Inc. (Jinju, Korea) and identified by Roy et al. (32). The IJs were multiplied using L5 larvae of *S. exigua*. Briefly, approximately 500 IJs in 200 µL distilled water were tropically applied to a Petri dish (9 cm diameter, 3 cm height) containing an individual larva. Treated larvae were incubated at the rearing conditions for 72 h. Dead larvae were transferred to a White trap (33) and IJs were harvested every day. These collected IJs were stored in sterilized distilled water at 10°C for no more than 21 days before use (34).

### Gut penetration assays

According to the method described by Roy and Kim (35), each well of a 6-well plate (Thermo Fisher Scientific Korea, Seoul, Korea) contained a piece of cabbage leaf (~1 cm^2^) on a paper towel and 80 IJs in 100 μL distilled water was applied on the leaf before adding L5 larva that had been starved for 6 h. The larvae completely ate the treated leaf piece in 30 min and were then incubated with a supply of nematode-free artificial diet at room temperature (RT) for predetermined time periods. The treated larvae were then rinsed in distilled water to remove any IJs on the outer surface and dissected longitudinally to isolate the intestines. Hemolymph and fat body in the open hemocoel were collected into 1 mL PBS and used for counting nematodes under a stereomicroscope (Stemi SV11, Zeiss, Jena, Germany) at 50× magnification (Marienfeld, Lauda-Konigshofen, Germany). Nematodes in the gut lumen were also collected for counting as described above. Three larvae were used in each measurement and replicated three times per treatment.

### RNA extraction, cDNA construction, and RT-qPCR

Total RNAs were extracted from control or nematode-treated larvae using Trizol reagent (Invitrogen, Carlsbad, CA, USA) according to the manufacturer’s instructions. Extracted RNAs were dissolved in 50 µL of diethyl pyrocarbonate-treated deionized and distilled water. Their concentrations were quantified with a spectrophotometer (Nanodrop, Thermo Fisher Scientific Korea). First-strand cDNAs were synthesized using ~1 µg of total RNA and Maxime RT PreMix (Intron Biotechnology, Seoul, Korea) containing oligo dT primer according to the manufacturer’s instruction. Synthesized cDNAs were used as templates for quantitative PCRs (qPCRs), which were performed using a real-time PCR machine (Step One Plus Real-Time PCR System, Applied Biosystem, Singapore) using Power SYBR Green PCR Master Mix (Life Technologies, Carlsbad, CA, USA) under the guideline of Bustin et al. (36). The reaction mixture (20 µL) contains 10 µL of PCR Master Mix, 5 µL of deionized distilled water, 3 µL of cDNA template (60 ng/µL), and 1 µL each of forward and reverse primers (**S1 Table**). The temperature program for qPCR began with 95°C heat treatment for 10 min followed by 40 cycles of denaturation at 94°C for 30 s, annealing at 52°C (*Se-Duox* and *Se-PGE_2_R*) or 58°C (Se-DSP1) for 30 s, and extension at 72°C for 30 s. The expression level of a ribosomal protein gene, *RL32*, was used for reference to normalize target gene expression levels. Quantitative analysis of transcripts followed the comparative CT (2^-ΔΔCT^) method (37).

### Reactive oxygen species measurement

An OxiSelect Intracellular ROS Assay Kit (Cell Biolabs, San Diego, CA, USA) was used to quantify ROS in L5 larvae of *S. exigua*. For measuring ROS level in the gut lumen, gut content was collected by squeezing out the midgut and centrifuged at 1,000 × *g* for 3 min. The supernatant was mixed with the same volume of 0.1× DCFH-DA (dichlorofluorescein diacetate). After incubation at 37°C for 30 min, the reaction product (150 μL) was then transferred to a 96-well plate and fluorescence was read at an emission wavelength of 530 nm after excitation at 480 nm. A calibration curve was drawn using serial dilutions of dichlorofluorescein standard in TC-100 cell culture medium. ROS levels were normalizing with protein amount (38). Each treatment was replicated three times with independent sample preparations using a single insect per replication.

### Bioassay of nematode and compounds against *S. exigua* larvae

For nematode treatment, 80 IJs/larva were used in the Petri dish assay (1 larva per dish). L5 larvae were used for all bioassays. During nematode treatment, test larvae were treated along with vitamin C (100 mg/mL), paraquat (25 µg/mL), or EMP. Briefly, fresh cabbage leaves (~1 cm^2^) were dipped into a test chemical solution for 5 min and dried under aseptic conditions. Then, 50 µL of sterile water containing 80 IJs was spread on the test chemical-treated leaves before the release of larvae. Mortality was assessed at 48 h for EMP assay and 72 h for vitamin C and paraquat assay after the nematode treatment. Each treatment was replicated three times with 10 larvae per replication.

### PLA_2_ activity

Secretory PLA_2_ (sPLA_2_) activity in plasma was fluorometrically determined using a commercial assay kit (sPLA_2_ Assay Kit, Cayman Chemical, Ann Arbor, MI, USA) with diheptanoyl thio-phosphatidyl choline as enzyme substrate by following the method described by Vatanparast et al. (39). Cellular PLA_2_ (cPLA_2_) activity measurement used the same kit except arachidonyl thio-phosphatidyl choline as the substrate. A spectrofluorometer (VICTOR multi-label Plate reader, PerkinElmer, Waltham, MA, USA) was used to measure enzyme activity. Changes in absorbance at 405 nm of the reaction product were measured and plotted to obtain the slope of a linear portion of the curve. Each treatment was replicated with three biologically independent enzyme preparations using different samples. Specific enzyme activity (µmol/min/µg) was calculated by dividing absorbance change by the protein amount used as the enzyme source for the reaction.

### Western blotting

For nematode treatment, L5 larvae were fed with nematode (80 IJs/larva)-treated cabbage (~1 cm^2^) for 8 h. For the inhibitory assay, the leaves were dipped into EMP (1,000 ppm) and dried before nematode (80 IJs/larva) treatment. At 8 h post treatments, hemolymph was collected carefully into PBS containing protease inhibitor cocktail (Sigma-Aldrich Korea) and 1 mM phenylmethylsulfonyl fluoride (Thermo Fisher Scientific Korea). For non-pathogenic bacterial challenges, L5 larvae were fed with *Escherichia coli* Top10 (2.2×10^4^ cells/mL) and *Pantoea agglomerans* ANU101 (1.9×10^4^ cells/mL) treated cabbage (~1 cm^2^). For pathogenic microorganisms, *Metarhizium rileyi* (1,000 conidia/mL), *Bacillus thuringiensis* subsp. *aizawai* (BtA, 1.3×10^4^ spores/mL), and a baculovirus (SeMNPV, 3.6×10^3^ plaque-forming unit/mL) were applied to cabbage leaves for feeding. Hemolymph samples were collected at 0-48 h PI. Using centrifugation at 500 × g for 5 min, plasma was separated from hemolymph and mixed with 4× denatured sample buffer (200 mM Tris-HCL, 400 mM DTT, 8% SDS, 0.4% bromophenol blue, and 40% glycerol). After 5 min at 95°C denaturation, proteins (20 µg per sample) were separated on 10% SDS-PAGE at 95 V. Separated proteins from the gel were transferred onto a nitrocellulose membrane (BioRad, Hercules, CA, USA) for 50 min at 95 V in chilled transfer buffer (25 mM Tris, 190 mM glycine, 20% methanol, pH 8.5). Membranes were briefly rinsed with Tris-buffered saline containing Tween-20 (TBST) (20 mM Tris, 150 mM NaCl, and 0.1% Tween 20, pH 7.5) and then blocked with 3% bovine serum albumin (BSA) in TBST at RT for 1 h. Membranes were then subjected to incubation with DSP1 antibody raised against Se-DSP1 in rabbit (Abclone, Seoul, Korea) diluted 5,000 times with TBST containing 3% BSA at 4°C for overnight. Following three washing with TBST (10 min for each), the membrane was incubated with an anti-rabbit IgG-alkaline phosphatase secondary antibody (Sigma-Aldrich, Korea) at a dilution of 1: 20,000 in TBST containing 3% BSA for 1 h at RT. Blots were rinsed three times with TBST. α-tubulin was used as a reference protein and detected by a polyclonal antibody (GTX112141; GeneTex, Irvine, CA, USA) after 1,000 times dilution. To detect alkaline phosphatase activity, nitrocellulose membrane blots were incubated with a substrate (BICP/NBT, Sigma-Aldrich Korea).

### Immunofluorescence assay

L5 larvae were fed with nematode (80 IJs/larva)-treated cabbage leaves. After 8 h feeding, the midgut was collected on a slide glass. After removing gut contents, TC-100 insect tissue culture medium was added immediately and incubated in a wet chamber for 5 min. After removing the medium, the gut sample was then fixed with 4% formaldehyde for 20 min at RT. The fixative was replaced with PBS and incubated for 10 min. Following two more washings with PBS, the midgut was permeabilized with 0.2% Triton X-100 in PBS for 20 min at RT. Following three washings with PBS, blocking was done with 5% skimmed milk powder in PBS for 20 min. A DSP1 antibody (20) raised against Se-DSP1 in rabbit was added after being diluted in 3% BSA in PBS (1:100) and incubated for 1 h 20 min at RT. After three washing with PBS, midgut was incubated with anti-rabbit secondary antibody (Sigma-Aldrich Korea) diluted in 3% BSA in PBS (1:5,000) for 1 h. After three washings with PBS, midguts were incubated for 5 min with 4′,6-diamidino-2-phenylindole (DAPI, 1 μg/mL) (Thermo Scientific Korea) in PBS for nucleus staining. For detection of PGE2 level in gut epithelium, 1% of PGE_2_ antibody (ab2318, Abcam, Cambridge, UK) was used in PBS after blocking with 5% skimmed milk at RT for 2 h. After washing three times with PBS, 1% anti-rabbit-FITC conjugated antibody (Thermo Fisher Scientific Korea) was used in PBS at RT for 1 h. After washing three times, the nucleus was stained with DAPI (1 mg/mL) as described above. Finally, following three washings with PBS, the tissue samples were observed with an addition of 50% glycerol and cover glass under a fluorescence microscope (DM2500, Leica, Wetzlar, Germany) at 200× magnification.

### Construction of double-stranded RNA and RNA interference

Double-stranded RNAs (dsRNAs) specific to *Se-DSP1* or *Se-PGE_2_R* were prepared as described previously (14). Briefly, DNA fragments were obtained by PCR using gene-specific primers (**S1 Table**) containing the T7 promoter sequence at the 5’ end. The PCR product was used as a template to generate dsRNA using a MEGAscript RNAi kit (Ambion, Austin, TX, USA) according to the manufacturer’s instructions. Sense and antisense RNA strands were synthesized using T7 RNA polymerase at 37°C for 4 h. A control dsRNA (dsCON) was also prepared by synthesizing 520 bp fragment dsRNA of CpBV302, a viral gene. The resulting dsRNA was purified and mixed with transfection reagent Metafectene PRO (Biontex, Plannegg, Germany) at a ratio of 1:1 (v/v) followed by incubation at 25°C for 30 min to form liposomes. One microgram of dsRNA was injected into larval hemocoel (1-day-old L5 larvae) using a microsyringe (Hamilton, Reno, NV, USA) equipped with a 26 gauge needle. At 12, 24, 48, and 72 h post-injection (PI), RNAi efficacies were determined using RT-qPCR as described above.

### *In vitro* Ca^2+^ signaling in response to nematode

L5 larvae were challenged with nematode (80 IJs/larva) for 8 h. Midgut tissues of the challenged larvae were dissected carefully in TC-100 insect medium and incubated in a wet chamber for 10 min. After removing TC-100, it was then incubated with 3 µL Fura-8AM (1 mM) in RT for another 10 min. Then midgut was washed three times with PBS and midgut cells were permeabilized with 0.2% Triton X-100 in PBS for 20 min at RT. Following washing three times with PBS, the midgut was incubated for 5 min with DAPI (1 μg/mL) in PBS for staining the nucleus. Finally, the midgut was washed three times with PBS and fixed with 50% glycerol (Duksan, Seoul, Korea). Fura signals in the midgut were observed under a fluorescence microscope (DM500, Leica, Wetzlar, Germany) at 200× magnification. Fluorescence changes were analyzed by using ImageJ software (https://imagej.nih.gov/ij). Each treatment was replicated three times with independent sample preparations.

### Inhibition of Ca^2+^ signaling

L5 larvae were injected with 1 µL of DAN (1 µg/µL), 2-APB (1 µg/µL), or U-73122 (1 µg/µL) along with nematode (80 IJs/larva). After 8 h incubation, midgut preparation and staining of treated larvae were done as described above. Fura signals in the midgut tissues were observed under a fluorescence microscope (DM500, Leica, Germany) at 200× magnification. Each treatment was replicated three times with independent gut sample preparations.

### Statistical analysis

All bioassays were conducted in three independent biological replicates and plotted by mean ± standard error using a Sigma plot. Means and variances of treatments were compared by a least squared difference (LSD) test of one-way analysis of variance (ANOVA) using PROC GLM of SAS program (40) and discriminated at Type I error = 0.05.

## Results

### ROS produced by the catalytic activity of se-duox defends against the nematode infection in the midgut

**Figure 1:**
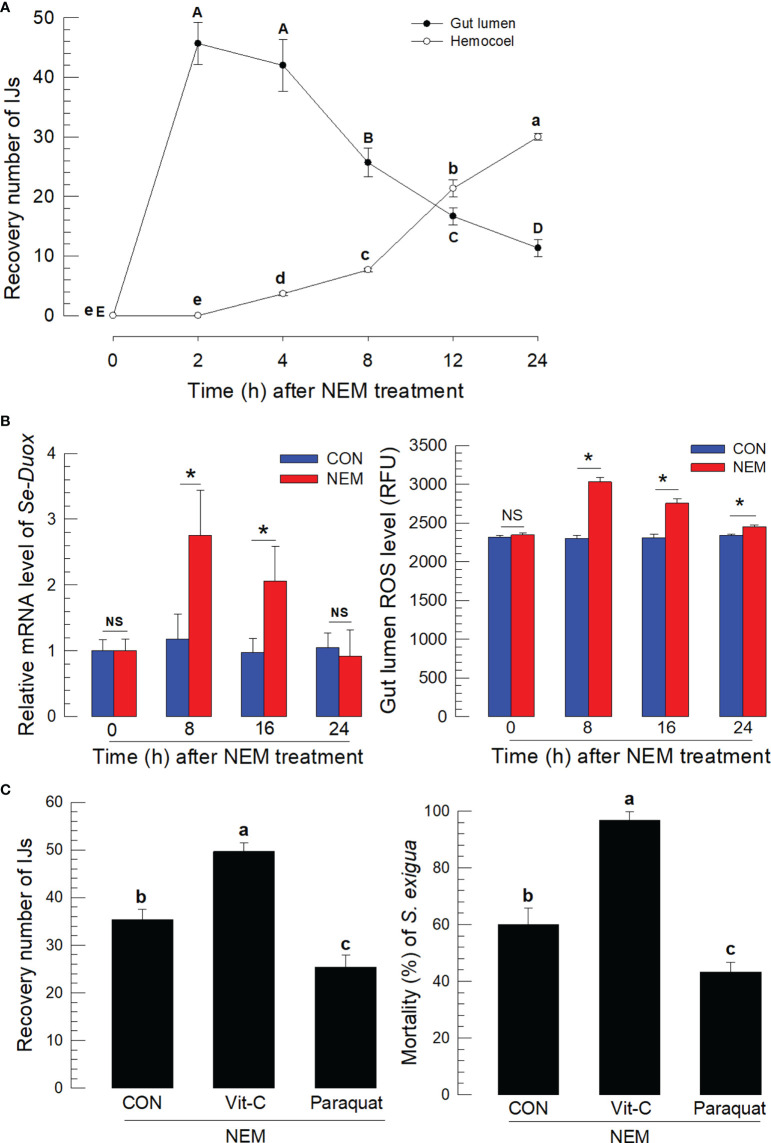
Entomopathogenic nematode (‘NEM’) infection to *S. exigua* gut leads to ROS burst. Each L5 larva of *S. exigua* was fed with 80 IJs of *S. feltiae*. **(A)** Temporal IJ infection from gut lumen to hemocoel after oral feeding treatment. **(B)** Induction of *Se-Duox* expression and ROS level. **(C)** Modulation of ROS levels against the nematode infection and subsequent virulence to *S. exigua*. L5 larvae were infected with IJs at 24 h after vitamin C (‘Vit-C’, 100 mg/larva) and paraquat (25 µg/larva) treatment. Larval mortality was recorded 72 h after the nematode treatment. Each treatment was replicated three times. Different letters and asterisks above standard error bars indicate significant differences among means at Type I error = 0.05 (LSD test). NS, no significance.

IJs of *S. feltiae* were fed to *S. exigua* using diet. The IJs in the midgut lumen infected the gut epithelium and entered the hemocoel (**Figure 1A**). A number of residual IJs in the gut lumen gradually decreased after 4 h following feeding treatment while the IJ number entering the hemocoel increased. The nematode infection to the gut epithelium was also monitored by measuring *Se-Duox* expression of the gut tissues and ROS amount in the gut lumen (**Figure 1B**). Significant (*P* < 0.05) increases in the gene expression and ROS production were observed in the larvae treated with the nematodes at 8~12 h after feeding treatment, which was the time for the active nematode infection to the midgut. To assess the effect of ROS on the nematode infection, vitamin C to suppress ROS and paraquat to induce ROS production were fed to the larvae along with the nematodes (**Figure 1C**). Vitamin C treatment significantly (*P* < 0.05) suppressed the ROS amount in the gut lumen in a dose-dependent manner (**Figure S1**) and increased the number of nematodes entering the hemocoel. In contrast, the paraquat treatment increased the ROS level and decreased the number of nematodes entering the hemocoel. The regulation of ROS amounts in the gut lumen by vitamin C or paraquat treatment significantly (*P* < 0.05) influenced the nematode virulence, in which vitamin C treatment increased the insecticidal activity of the nematode while paraquat treatment decreased.

### DSP1 mediates the nematode infection signal in the midgut

**Figure 2:**
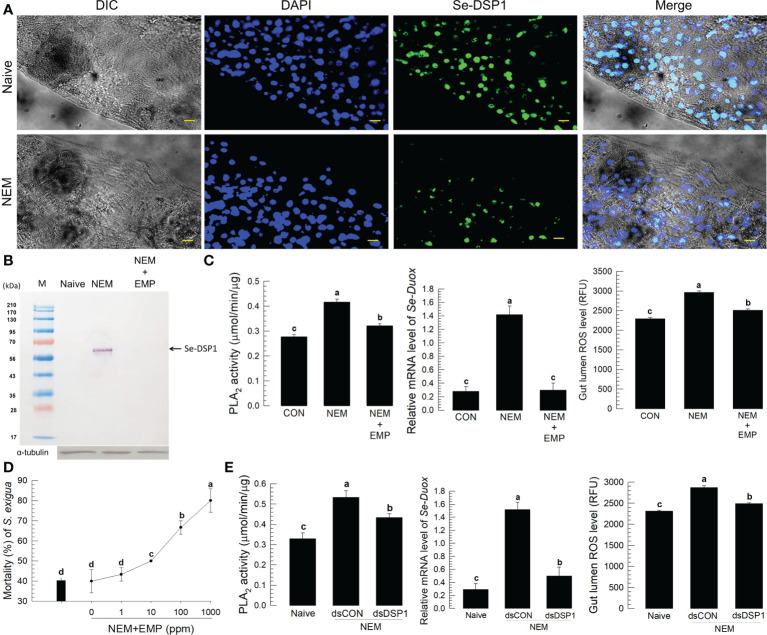
Release of Se-DSP1 from the midgut epithelium to defend the entomopathogenic nematode (‘NEM’) infection. Each L5 larva of *S*. *exigua* was fed with 80 IJs of *S*. *feltiae*. **(A)** Nuclear localization of Se-DSP1 in the midgut epithelium of *S*. *exigua*. An immunofluorescence image was obtained by staining Se-DSP1 using a specific antibody labeled with FITC and nuclei with DAPI. ‘DIC’ represents differential interference contrast. The scale bar represents 10 µm. **(B)** A western blotting showing release of Se-DSP1 upon the nematode infection into plasma, which was obtained 8 h after the nematode treatment. 3-Ethoxy-4-methoxyphenol (‘EMP’, 1,000 ppm) was treated along with the nematodes. α-Tubulin was stained as a control to confirm the equal amount of protein loading. **(C)** Effect of Se-DSP1 release on PLA_2_ activity in plasma, *Se-Duox* expression in the epithelium, and ROS level in the gut lumen. **(D)** Enhancement of the nematode (80 IJs/larva) virulence with the addition of EMP against L5 larvae of *S*. *exigua*. Mortality was assessed at 48 h after the nematode treatment. **(E)** Effect of PLA_2_ activity induced by Se-DSP1 release on *Se-Duox* expression and ROS production. RNAi was performed by injecting dsRNA (1 µg) specific to *Se-DSP1*. The nematode treatment was followed at 24 h after the dsRNA injection. At 8 h after the nematode treatment, *Se-Duox* expression and ROS production were assessed. Each treatment was replicated three times. Different letters above the standard error bars indicate significant differences among means at Type I error = 0.05 (LSD test).

To assess the interaction between the nematode infection and the insect midgut epithelium, a damage-associated molecular pattern (Se-DSP1) was analyzed in the midgut of *S. exigua* after the nematode infection (**Figure 2**). One cell layer of the midgut epithelium was observed under a fluorescence microscope, in which Se-DSP1 was localized in the nucleus stained with DAPI (**Figure 2A**). However, Se-DSP1 was released to hemolymph after the nematode infection to the midgut epithelium, in which the control hemolymph collected from the naïve larvae did not have the corresponding band in the western blot (**Figure 2B**). The nematode treatment also significantly increased PLA_2_ activity in the midgut epithelium (**Figure 2C**) as well as in the plasma (**Figure S2**). However, when a specific inhibitor (3-ethoxy-4-methoxyphenol: EMP) to Se-DSP1 (20) was applied along with the nematode, the release of Se-DSP1 from the nucleus to hemolymph was not detected. This EMP treatment also suppressed the induction of PLA_2_ activity after the nematode treatment and prevented the induction of *Se-Duox* expression and subsequent ROS production even in response to the nematode infection. The additional EMP significantly increased the nematode virulence in a dose-dependent manner (**Figure 2D**). The modulation of PLA_2_ activity/*Se-Duox* expression/ROS production by Se-DSP1 was further supported by the RNAi specific to Se-DSP1. Specific dsRNA injection suppressed the Se-DSP1 expression (**Figure S3**) and prevented its induction of PLA_2_ activity (**Figure 2E**). The dsRNA treatment also suppressed *Se-Duox* expression and subsequently presented the up-regulation of ROS level in the gut lumen. These indicate an immune signaling pathway of DSP1/PLA_2_/Duox after the nematode infection in the midgut of *S. exigua*.

### PGE_2_ induces Ca^2+^ level to activate duox for ROS production

**Figure 3:**
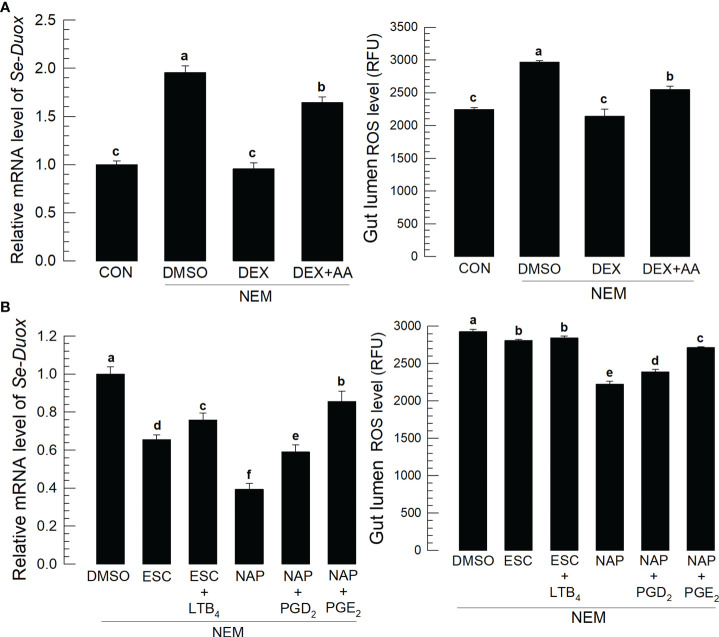
Effects of eicosanoids on expression levels of *Se-Duox* and ROS production in response to gut infection by the entomopathogenic nematode (‘NEM’). Each L5 larva of *S*. *exigua* was fed with 80 IJs of *S*. *feltiae*. **(A)** Inhibitory effect of a PLA_2_ inhibitor, dexamethasone (‘DEX’), on *Se-Duox* expression and ROS level in the gut lumen of L5 larvae. NEM treatment was performed 8 h post-injection (PI) of DEX (10 µg/larva) or arachidonic acid (AA, 10 µg/larva). At 8 h after NEM treatment, *Se-Duox* expression and ROS level were measured. **(B)** Effect of naproxene (‘NAP’, a COX inhibitor) and esculetin (‘ESC’, a LOX inhibitor) on the expression of *Se-Duox* and ROS level. To rescue the inhibitor treatments, LTB_4_, PGD_2,_ or PGE_2_ was injected at 1 μg/larva along with ESC or NAP treatment. Each treatment was replicated three times. Different letters above the standard error bars indicate significant differences among means at Type I error = 0.05 (LSD test).

Induction of PLA_2_ activity by Se-DSP1 after the nematode infection suggested the production of eicosanoids, which would induce *Se-Duox* expression for ROS production. **Figure 3A** supported the role of PLA_2_ for eicosanoid biosynthesis because a specific inhibitor, dexamethasone (DEX), to PLA_2_ significantly (*P* < 0.05) suppressed *Se-Duox* expression and ROS production. The regulation of PLA_2_ activity also altered the ROS amount in the midgut. An addition of arachidonic acid (‘AA’), a catalytic product of PLA_2_, significantly (*P* < 0.05) rescued the expression of *Se-Duox* and subsequent ROS production. To determine the type of eicosanoids, two different eicosanoid inhibitors, esculetin (a LOX inhibitor) and naproxen (a COX inhibitor), were administered to the larvae infected with the nematode (**Figure 3B**). Both inhibitors significantly suppressed *Se-Duox* induction and ROS production, in which the COX inhibitor was much more potent. An addition of COX product (PGD_2_ or PGE_2_) significantly rescued *Se-Duox* induction and ROS production in the larvae treated with DEX, in which PGE_2_ was more active.

**Figure 4:**
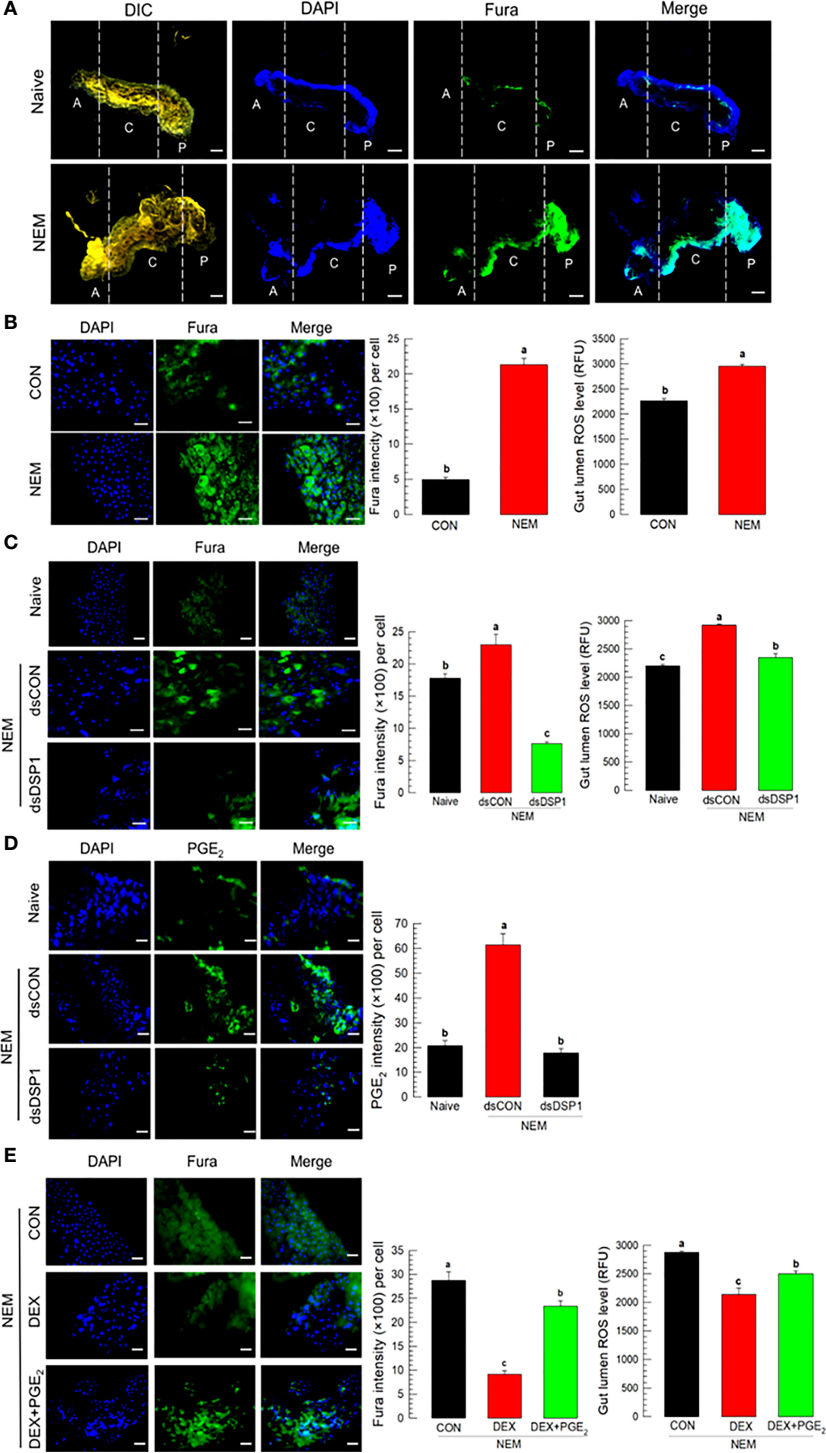
Ca^2+^ signal after Se-DSP1 release *via* PGE_2_ in response to gut infection by the entomopathogenic nematode (‘NEM’). Each L5 larva of *S*. *exigua* was fed with 80 IJs of *S*. *feltiae*. **(A)** Ca^2+^ signals in the entire gut are separated into anterior (‘A’), central (‘C’), and posterior (‘P’). Nuclei were stained with DAPI. Fura-8AM was used to observe Ca^2+^. ‘DIC’ represents differential interference contrast. The scale bar indicates 100 µm. **(B)** Induction of Ca^2+^ signal and subsequent up-regulation of ROS level in midgut in response to NEM treatment for 8 h. **(C)** Effect of RNAi specific to *Se-DSP1* expression on Ca^2+^ signal in response to NEM treatment. At 24 h after dsRNA (1 µg/larva) injection, NEM was treated. After 8 h NEM treatment, Ca^2+^ level and ROS amount were measured. **(D)** Induction of PGE_2_ in the midgut epithelium. **(E)** Inhibitory effect of a specific PLA_2_ inhibitor (dexamethasone (DEX) on Ca^2+^ signal and ROS level in midgut. NEM treatment was performed at 8 h after injection of DEX (10 µg/larva) or PGE_2_ (1 µg/larva). Nuclei were stained with DAPI. PGE_2_ was detected with a specific antibody raised against rabbit. Fura-8AM was used to observe Ca^2+^. Scale bars in B-E represent 10 µm. Different letters above standard error bars indicate significant difference among means at Type I error = 0.05 (LSD test).

Nematode infection up-regulated Ca^2+^ level in the midgut epithelium, in which the Ca^2+^ signal was observed with a fluorescent calcium chelator, Fura-8AM (**Figure 4**). The Ca^2+^ signal was more prominent in the central and posterior regions than in the anterior region of the midgut (**Figure 4A**). Subsequent Ca^2+^ signal assays used the central region of the midgut. The induction of Ca^2+^ signal by the nematode treatment was recorded by more than 4 folds, which was followed by an increase of ROS amount in the gut lumen (**Figure 4B**). However, RNAi specific to *Se-DSP1* expression prevented the induction of the Ca^2+^ signal and subsequent ROS production (**Figure 4C**). The nematode treatment also significantly (*P* < 0.05) increased the PGE_2_ level in the midgut epithelium, which was, however, prevented by the RNAi treatment specific to *Se-DSP1* expression (**Figure 4D**). Addition of PGE_2_ to larvae treated with DEX rescued the inhibition of the induced Ca^2+^ signal and subsequent ROS production (**Figure 4E**).

### PGE_2_ induces Ca^2+^ signal *via* its specific receptor and subsequent IP_3_ signal pathway to upregulate Ca^2+^ level

**Figure 5:**
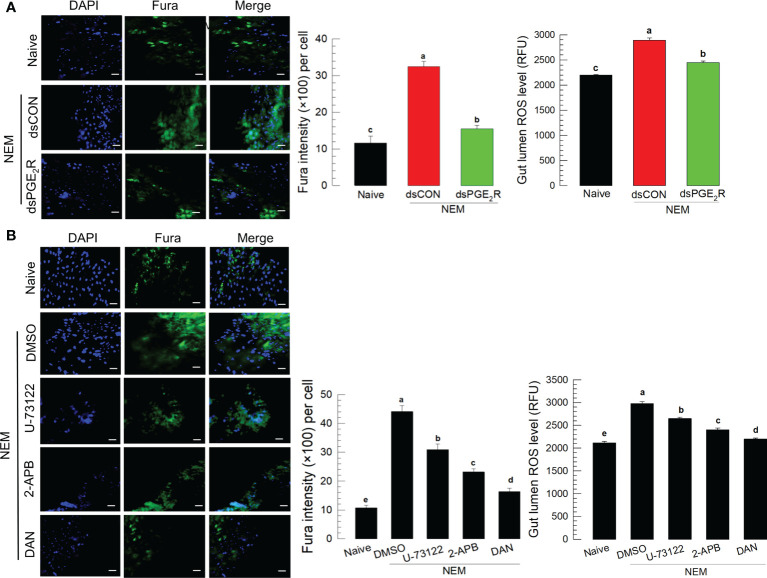
Effect of PGE_2_ receptor (‘PGE_2_R’) and its downstream signal on the induction of Ca^2+^ signal in response to gut infection by the entomopathogenic nematode (‘NEM’) infection. Each L5 larva of *S*. *exigua* was fed with 80 IJs of *S*. *feltiae*. **(A)** Effect of RNAi against *Se-PGE_2_R* expression on calcium signal and ROS production. NEM treatment was performed at 24 h after dsRNA (1 µg/larva) injection. **(B)** Inhibition of Ca^2+^ signal by four different calcium signal inhibitors: dantrolene sodium (‘DAN’, a specific inhibitor to ryanodine receptor), 2-APB (a specific IP_3_ receptor inhibitor), U-73122 (a specific PLC inhibitor) along with NEM treatment. All inhibitors were injected in a dose of 1 µg per larva. The guts of treated larvae were dissected at 8 h after the NEM or inhibitor treatment. Nuclei were stained with DAPI. Fura-8AM was used to observe Ca^2+^. Each treatment was replicated three times. The scale bar represents 10 µm. Different letters above standard error bars indicate significant difference among means at Type I error = 0.05 (LSD test).

To support the role of PGE_2_ in mediating gut immunity to produce ROS, its specific receptor, *Se-PGE_2_R* expression was suppressed by RNAi for a loss-of-function approach (**Figure 5A**). The RNAi treatment significantly suppressed its expression levels for at least 72 h (**Figure S4**). The RNAi suppressed the induction of Ca^2+^ level in the gut epithelium and subsequent ROS amount in the gut lumen after the nematode treatment. The downstream signaling pathway of PGE_2_R was then assessed with specific inhibitors along with the nematode treatment (**Figure 5B**): sodium dantrolene (‘DAN’) against ryanodine receptor (‘RyR’), 2-APB against IP_3_ receptor (‘IP_3_R’), and U-73122 against PLC. All these inhibitors significantly (*P* < 0.05) suppressed the induction of Ca^2+^ level in the gut epithelium and subsequent ROS amount in the gut lumen.

### DSP1 release only at pathogenic infections

**Figure 6:**
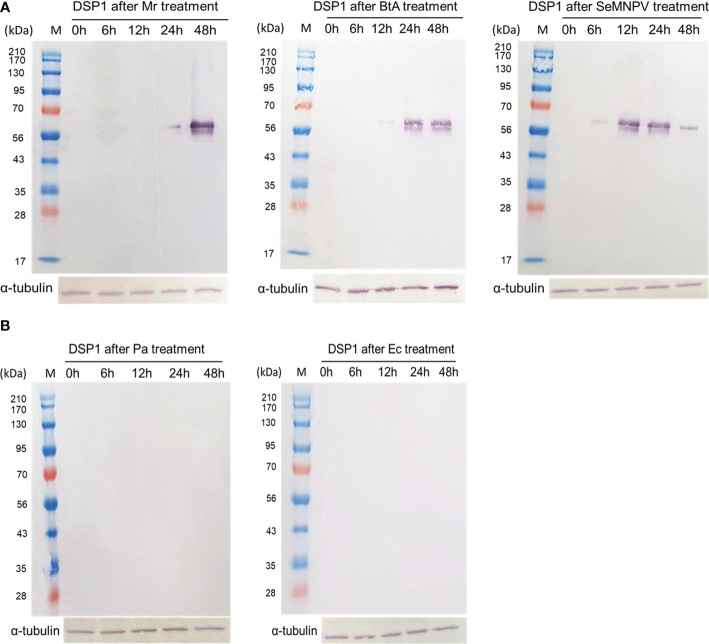
Release of Se-DSP1 into plasma upon infection of different microbes. Western blotting shows the release of Se-DSP1 into plasma upon gut infection by **(A)** entomopathogenic fungus, *M*. *rileyi* (‘Mr’), entomopathogenic bacterium, *B. thuringiensis* subsp. *aizawai* (‘BtA’), a baculovirus (‘SeMNPV’), and **(B)** non-pathogenic bacteria, *E. coli* (‘Ec’) and *P*. *agglomerans* (‘Pa’). Hemolymph was collected and plasma was separated at 0-48 h PI. Se-DSP1 antibody was used to detect DSP1 in plasma where α-tubulin was used as a reference to confirm the equal amount of protein loading.

To support the DAMP role of DSP1, *S. exigua* larvae were challenged with other pathogenic and non-pathogenic microbes (**Figure 6**). Pathogenic microbes used a fungal *M. rileyi*, a bacterial *B. thuringiensis*, and a baculoviral SeMNPV. Non-pathogenic microbes used two enterobacteria, *E. coli*, and *P. agglomerans*. After oral feeding, plasma samples were collected from the test larvae. DSP1 was released into plasma during pathogenic infections, in which it began to be released as early as 6 h for the viral infection. However, DSP1 was not released at non-pathogenic infections for 48 h after treatment.

## Discussion

Gut immunity using ROS and antimicrobial peptides is triggered after recognition of the presence of harmful microbes among the gut microbial flora to remove pathogens in the gut lumen (41, 42). However, the pathogen recognition by the gut epithelium remained unclear though bacterial-derived uracil has been regarded as a pathogen signal (7). This study showed a general damage signal molecule, HMGB1-like DSP1, as a pathogen-associated molecule triggering the gut immunity in a lepidopteran insect, *S. exigua*, against the nematode infection.

The nematode infection using *S. feltiae* was fatal to *S. exigua via* oral administration in this study. *S. feltiae* has been used to control diverse insect pests because of its wide host spectrum (43). Indeed, *S. exigua* was an optimal host for investigating *S. feltiae* pathogenicity because IJs exhibited higher virulence and produced more progeny IJs in *S. exigua* compared to other susceptible insects, *Tenebrio molitor* because the hemocoelic penetration rate of the IJs from the gut lumen to hemocoel was faster with more numbers in *S. exigua* than *T. molitor* 24 h after feeding treatment (35). In our current study, *S. feltiae* began to infect the hemocoel 4 h after feeding treatment and continued the hemocoelic infection until 24 h, during which ROS amount in the gut lumen increased along with induction of *Se-Duox* expression. This ROS production was confirmed to be protective against the nematode infection because vitamin C (= ROS quencher as an antioxidant) treatment significantly increased insect mortality, while paraquat (= ROS producer) treatment significantly suppressed the nematode virulence. Otherwise, the IJs entering hemocoel release the symbiotic *Xenorhabdus bovienii* to kill the host insect by inducing septicemia (32). This supports the role of gut immunity using ROS in defending the nematode infection in *S. exigua*.

The concomitance of the nematode infection and ROS production suggests a gut damage signal operating during the nematode infection to *S. exigua* gut. DSP1 is an insect homolog of vertebrate HMGB1 that acts as a damage-associate molecular pattern (DAMP) in the extracellular matrix as well as its transcriptional regulator in the nucleus (44). In vertebrates, HMGB1 is released from the nucleus into plasma under immune challenge and interacts with specific receptors such as Toll-like receptors to activate immune responses (28, 45). In *S. exigua*, DSP1 is identified and known to be released from hemocytes or fat body under immune challenge (20). The released DSP1 in plasma activates Toll receptors to induce cellular and humoral immune responses in *S. exigua* (46). In our current study, DSP1 was observed in the nucleus of the gut epithelium of *S. exigua*. Under the nematode infection by oral feeding, DSP1 was observed in plasma while none was detected in the plasma from naïve larvae. This suggests that DSP1 in the nucleus of the gut epithelium was released to plasma after the gut damage induced by the nematode infection. Interestingly, the DSP1 release was observed only in infection with pathogenic microbes, but not with non-pathogenic microbes. This suggests that DSP1 release is specific to pathogens in the gut and plays a recognition role in discriminating harmful microbes from commensal or mutualistic microbes.

The release of DSP1 up-regulated PLA_2_ activity and ROS amount in the gut lumen along with induction of *Se-Duox* expression because the treatment of 3-ethoxy-4-methoxyphenol (EMP) inhibiting DSP1 release from the nucleus (46) prevented the subsequent activating processes. The up-regulation of PLA_2_ activity after the DSP1 release induced by the nematode infection led to an increase of PGE_2_ in the gut epithelium of *S. exigua*. This supports our previous study showing the direct activation of PLA_2_ by DSP1 using an injection of a recombinant DSP1 to hemocoel (20). PLA_2_ catalyzes phospholipids to release arachidonic acid, which is then oxygenated to various eicosanoids (47). Prostaglandins (PGs) are produced by a specific oxygenase susceptible to naproxen (48). The induction of *Se-Duox* expression or subsequent ROS production was significantly inhibited by the PG-specific inhibitor while the addition of PGD_2_ or PGE_2_ significantly rescued the inhibition. Both PGs are known to be present in different larval tissues of *S. exigua* (49). Between these two PGs, PGE_2_ was superior in the rescuing activity to PGD_2_ at the same dose (1 µg/insect). Indeed, the increase in PGE_2_ level was observed in the gut epithelium after the nematode infection. Especially, the up-regulation of PGE_2_ in the gut epithelium was highly dependent on DSP1 because a specific RNAi against *Se-DSP1* expression suppressed the increase of PGE_2_ level. Furthermore, PGE_2_ alone up-regulated ROS production. These suggest that PGE_2_ is required for ROS production in the gut epithelium in response to the nematode infection.

**Figure 7:**
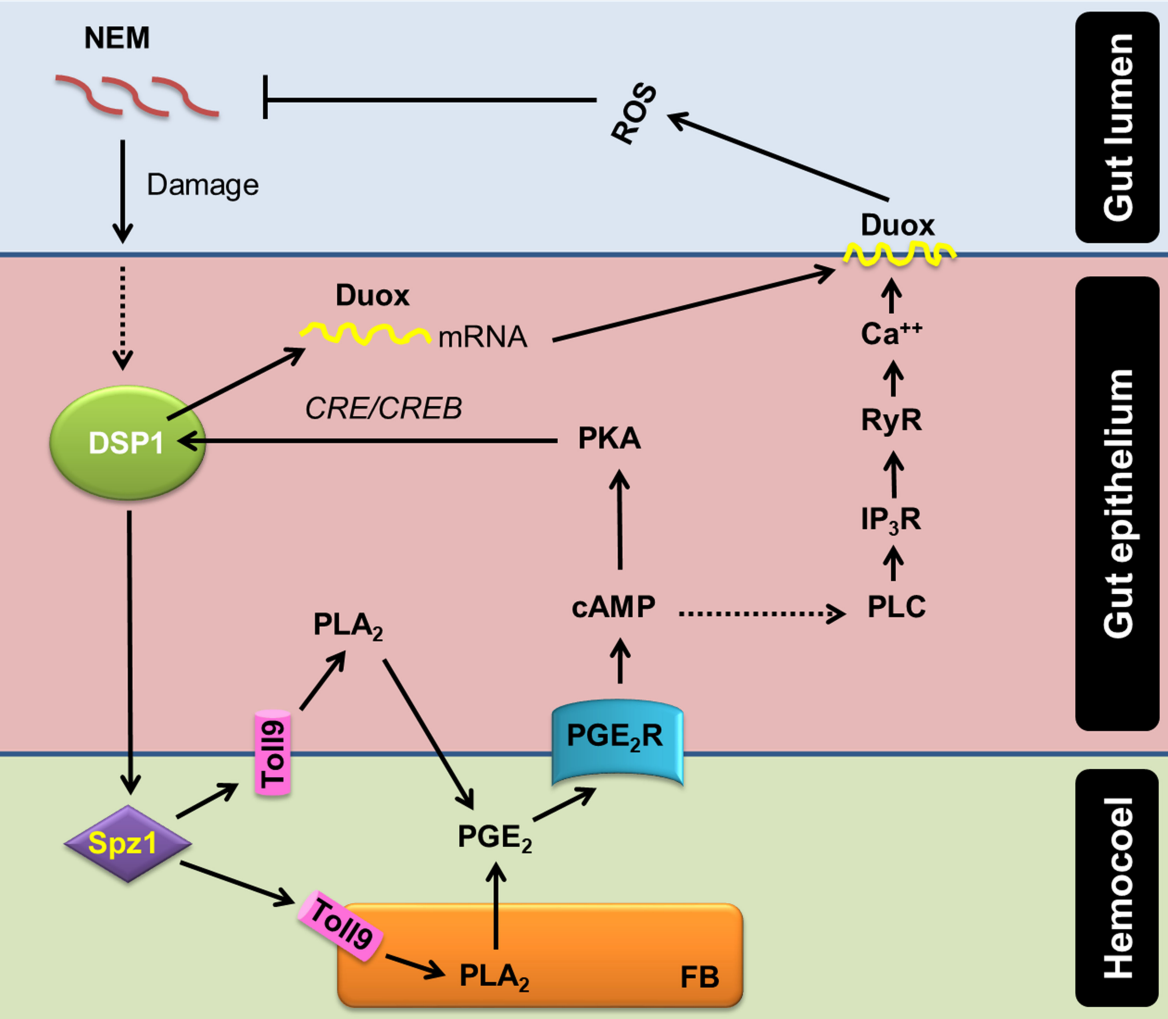
A model of PGE_2_ mediating DSP1/Ca^2+^/Duox signaling pathway in the midgut epithelium of *S*. *exigua* in response to an entomopathogenic nematode (‘NEM’) infection. Upon infection of gut epithelium by EPN, a damage-associated pattern molecule (‘DSP1’) is released to hemocoel. Released DSP1 binds to spätzle 1 (‘Spz1’) to activate the Toll9 receptor to produce PGE_2_ in the gut epithelium or fat body (‘FB’) by activating phospholipase A_2_ (‘PLA_2_’) [20]. The autocrine/paracrine PGE_2_ binds to its specific receptor (‘PGE_2_R’) to up-regulate cAMP, which triggers *Se-Duox* expression *via* PKA/CREB [15]. cAMP also activates phospholipase C (‘PLC’) to increase the inositol triphosphate (‘IP_3_’) level [19]. IP_3_ binds to its receptor (‘IP_3_R’) on the endoplasmic reticulum to release Ca^2+^ [19]. The released Ca^2+^ triggers calcium-induced calcium release from the ryanodine receptor (‘RyR’), which leads to a Ca^2+^ burst [19]. The up-regulated Ca^2+^ then activates dual oxidase (‘Duox’) to produce reactive oxygen species (‘ROS’) and finally defend NEM.

Ca^2+^ signal in the gut epithelium was significantly induced after the nematode infection. Especially, PGE_2_ mediated the increase of Ca^2+^ signal in the gut epithelium. The increase in Ca^2+^ signal led to an increase in ROS production in the gut lumen. PGE_2_ plays crucial roles in mediating various immune responses in insects (16). PGE_2_ receptor of *S. exigua* was identified (49) and elevated two secondary messengers, in which cAMP was earlier induced and required for the subsequent induction of Ca^2+^ signal (19). RNAi of PGE_2_ receptor gene expression significantly suppressed the Ca^2+^ signal up-regulated by the nematode infection in this current study. Subsequently, Se-PGE_2_ receptor up-regulated the Ca^2+^ signal by calcium-dependent calcium induction in the endoplasmic reticulum, in which IP_3_ and ryanodine receptors were required for the up-regulation of the Ca^2+^ signal in the gut epithelium in response to the nematode infection. Sajjadian et al. (15) add the role of cAMP in inducing *Se-Duox* expression *via* cAMP-responsive element (CRE)-binding protein (CREB) in response to PGE_2_ in the gut epithelium. Mollah et al. (20) showed that a specific combination of Späzle and Toll receptor (Spz1-Toll9) mediates PLA_2_ activation of DSP1. Altogether, our current results suggest a signal pathway of gut immunity from the nematode infection to ROS production using DSP1/PLA_2_/Ca^2+^/Duox (**Figure 7**).

Different damage signals have been proposed in insect gut immunity. In *Drosophila*, uracil derived from pathogenic bacteria can bind to unidentified G protein-coupled receptors and trigger the Gαq-PLCβ-Ca^2+^ pathway to activate MEKK1-MKK3-p38 MAPK with subsequent up-regulation of Duox gene expression or increased enzymatic activity through Ca^2+^-binding domain of Duox as mentioned in Introduction. In addition, lactic acid derived from *Lactobacillus* in the fly gut triggers ROS production, in which excessive ROS production ultimately damages the tissue of the gut epithelium leading to uncontrolled proliferation of intestinal stem cells and subsequent malfunctioning in the gut (50). However, it is little known how lactic acid induces ROS production. Our current study proposes an alternative explanation of how insect gut discriminates pathogenic microbes using an entomopathogenic nematode, which is not likely to produce uracil or lactic acid derived from bacteria. The direct penetration of the nematodes to the gut epithelium would give tissue damage, which releases DSP1 to hemocoel. In the hemocoel, the released DSP1 activates Toll signaling *via* its ligand Spätzle (46) on either gut epithelium or other tissues to activate PLA_2_ and the associated AMP production. Toll/IMD signaling pathways are closely associated with the activation of PLA_2_ activity in *S. exigua* and *Tribolium castaneum* (51, 52). The activated PLA_2_ produces eicosanoids, which up-regulated Duox gene expression and activity as demonstrated in two lepidopteran hosts, *S. exigua* and *Plutella xylostella* (14, 15). Among various eicosanoids, this current study showed that PGE_2_ is effective to induce Ca^2+^ signal and subsequent ROS production *via* Duox activation. PGE_2_ is produced in the midguts of different insects including *Manduca sexta* and *Helicoverpa zea* (two lepidopteran insects), *Periplaneta americana* (cockroach), and *Anopheles gambiae* (mosquito) (18, 53). In addition, DSP1 is conserved in most insect orders (30). These results propose a gut immune signal pathway of DSP1/PLA_2_/Ca^2+^/Duox in insects.

## Data availability statement

The original contributions presented in the study are included in the article/**Supplementary Material**. Further inquiries can be directed to the corresponding author.

## Author contributions

MR and YK carried out the experiment. MR and YK wrote the manuscript with support from SA. YK conceived the original idea. YK supervised the project. All authors contributed to the article and approved the submitted version.

## Funding

This work was supported by a grant (No. 2022R1A2B5B03001792) of the National Research Foundation (NRF) funded by the Ministry of Science, ICT and Future Planning, Republic of Korea and Korea Institute of Planning and Evaluation for Technology in Food, Agriculture, Forestry and Fisheries (IPET) through Exporting Promotion Technology Development Program funded by the Ministry of Agriculture, Food and Rural Affairs (MAFRA) (321100-3), Republic of Korea.

## Acknowledgments

We thank Duyeol Choi for his assistance in statistical analysis. We also appreciate Youngim Song for her supplying experimental materials.

## Conflict of interest

The authors declare that the research was conducted in the absence of any commercial or financial relationships that could be construed as a potential conflict of interest.

## Publisher’s note

All claims expressed in this article are solely those of the authors and do not necessarily represent those of their affiliated organizations, or those of the publisher, the editors and the reviewers. Any product that may be evaluated in this article, or claim that may be made by its manufacturer, is not guaranteed or endorsed by the publisher.
