# Supplementary material for: Dorsal switch protein 1 as a damage signal in insect gut immunity to activate dual oxidase via an eicosanoid, PGE2
Source: Front Immunol. 2022 Nov 10;13:994626. doi: 10.3389/fimmu.2022.994626 (PMC9691268; doi:10.3389/fimmu.2022.994626)
Supplement: Supplementary file 1 [file DataSheet_1.docx]

SUPPLEMENTARY MATERIAL

**Table S1.** Primers used in this study

| **Genes** | **Primer sequences (5' - 3')** | **Annealing temperature (^o^C)** | **Purposes** |
| --- | --- | --- | --- |
| *Se-DSP1* | CAAGCTGCATTACAACGGCA | 58 | RT-qPCR |
|  | CAGGGGACCCTTCTTGTACG |  |  |
|  | TAATACGACTCACTATAGGGAGACAAGCTGCATTACAACGGCA | 58 | RNAi |
|  | TAATACGACTCACTATAGGGAGACAGGGGACCCTTCTTGTACG |  |  |
| *Se-PGE_2_R* | AAGGCCCTTCCTCTACCAAA | 52 | RT-qPCR |
|  | AGATCATGAAGAGCACGGAG |  |  |
|  | TAATACGACTCACTATAGGGAGAAAGGCCCTTCCTCTACCAAA | 52 | RNAi |
|  | TAATACGACTCACTATAGGGAGAAGATCATGAAGAGCACGGAGAG |  |  |
| *Se-Duox* | AGGATCGTTATCGCTAGTTTACAG | 52 | RT-qPCR |
|  | GCCATCCCTAATAGGACTTCTTC |  |  |
| *RL32* | ATGCCCAACATTGGTTACGG | 52 | RT-qPCR |
|  | TTCGTTCTCCTGGCTGCGGA |  |  |


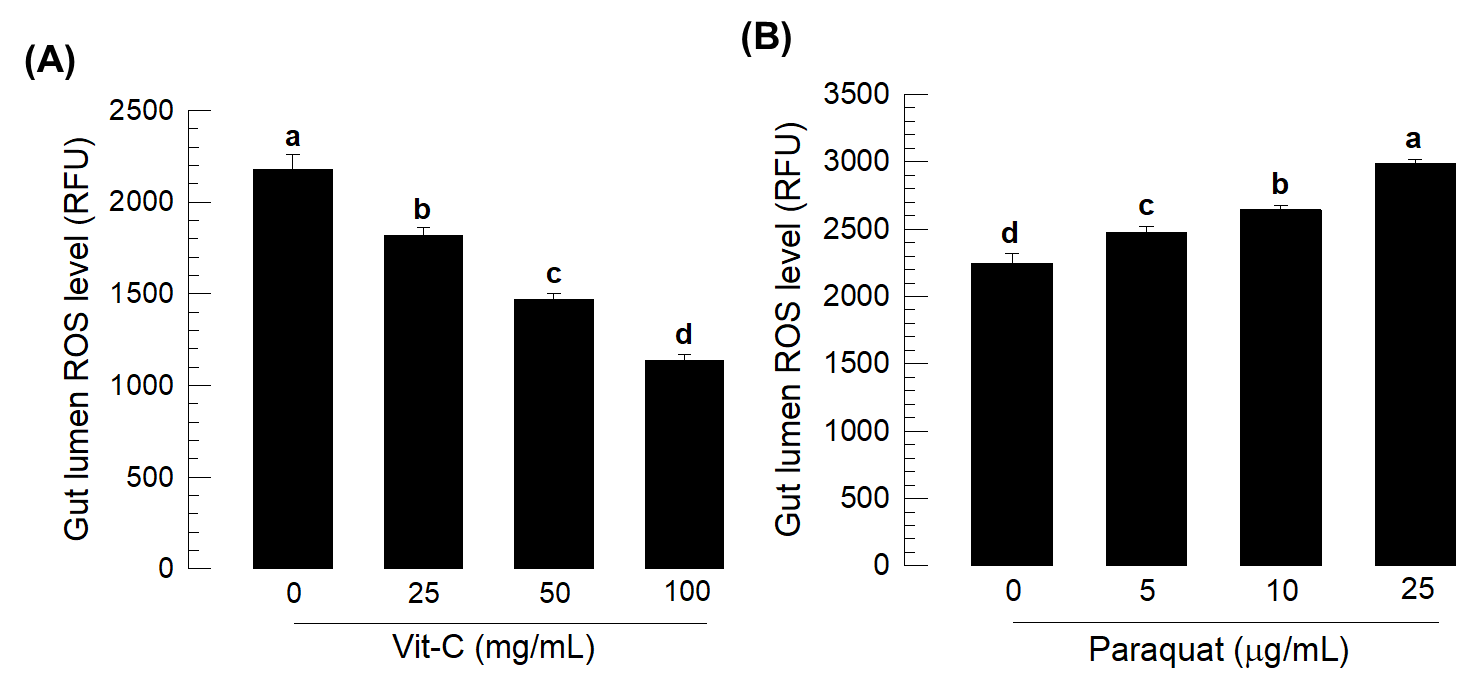


**Figure S1**

**Figure S2**

**Figure S3**

**Figure S4**
